# Assessing CREMAs’ Capacity to Govern Landscape Resources in the Western Wildlife Corridor of Northern Ghana

**DOI:** 10.1007/s00267-025-02155-9

**Published:** 2025-04-04

**Authors:** Eric Rega Christophe Bayala, Mathurin Zida, Kwabena Owusu Asubonteng, Mirjam A. F. Ros-Tonen, James Reed, Freddie Sayi Siangulube, Amy Ickowitz, Houria Djoudi, Terry Sunderland

**Affiliations:** 1 Center for International Forestry Research (CIFOR), Bogor, Indonesia; 2 Ministry of Environment, Water and Sanitation/Permament Secretariat for REDD+, Ouagadougou, Burkina Faso; 3 Department of Natural Resources and Geo-information Sciences, University for Development Studies (UDS), Tamale, Ghana; 4 Amsterdam Institute for Social Science Research (AISSR), University of Amsterdam, Amsterdam, The Netherlands; 5 Faculty of Forestry, University of British Columbia (UBC), Vancouver, Canada

**Keywords:** Landscape governance, Capacity, CREMA, Ghana, Conservation and development, Natural resource management

## Abstract

Ghana initiated community resource management areas (CREMAs) as a community-based natural management approach to give local communities the right and power to manage natural resources within their territorial boundaries. The expectation is that communities and their environment would prosper through more equitable landscape governance and sustainable use of natural resources. However, the challenges to achieving full functionality of CREMA and expected results, particularly in the Western Wildlife Corridor in northern Ghana, raise questions about the governance actors’ capacity. Therefore, this study aims to assess the capacity of actors to take ownership of and lead the governance processes implied by the CREMA approach. Based on focus group discussions and individual interviews, we found that the capacities of the CREMA governance bodies are weak to implement the CREMA approach effectively. The lack of knowledge and technical skills to support multi-actor processes, the weak collaboration between actors, and the lack of sustainable financial inflows and livelihood support are key challenges to be addressed for better CREMA performance. Despite these constraints, local actors’ enthusiasm and willingness to engage more actively in the governance of their landscape constitute an opportunity for an improved implementation of the CREMA approach. We suggest that initiatives to strengthen the technical and financial capacities of governance bodies and raise awareness among the local population are necessary to improve the functioning and performance of CREMAs. In addition, actions to improve the livelihoods of local communities will enhance the mobilization and engagement of social groups in the implementation of the CREMA concept.

## Introduction

The community-based natural resource management (CBRNM) approach has evolved in recent decades to promote more equitable governance of landscape resources (Adeyanju et al. 2021; Hajjar et al. 2021). In sub-Saharan Africa, it has been implemented to reconcile biodiversity conservation with poverty reduction by placing local communities at the core of natural resource governance (Clay 2016; Green 2016; Ouko 2018; Adeyanju et al. 2021). It aims to empower local people to manage their environments sustainably through participation and decentralization (Adeyanju et al. 2021).

In Ghana, the CBNRM approach is implemented through the CREMA (Community Resource Management Area) concept (Asare et al. 2013; Agyare et al. 2015; Foli et al. 2018; Wildlife Division 2000; Bayala 2024). The CREMA mechanism allows the national government to transfer rights and power to local communities (resource owners and users) to govern and manage forest and wildlife resources within a geographically defined area through the issuance of a devolution certificate (Agyare 2013; Asare et al. 2013). The CREMA concept is founded on democratic decision-making processes, allowing a group of communities to agree on the management regime of a common area for natural resource protection and economic and livelihood benefits (Asare et al. 2013; Bayala 2023). The CREMA is thus recognized both as a geographically defined area and as a community-based organization with elected executives responsible for its operation. Unless stated otherwise in a CREMA constitution, CREMA members generally refer to all residents—natives, and non-natives, including admitted members—within the defined boundaries of the CREMA, whilst the CREMA executives refer to all the democratically elected members of the CREMA governance bodies known as Community Resource Management Committees (CRMCs) and CREMA Executive Committee (CEC) (Agyare 2013; Agyare et al. 2015). The CEC is the top body that coordinates and supervises CREMA actions at the landscape level, while the CRMC serves as the CREMA decision-making body at the local level to carry out CREMA activities and ensure the application of laws governing the use of natural resources in the communities. The CRMC represents all social groups in the community (Agidee 2011; Asare et al. 2013; Bayala et al. 2024; Foli et al. 2018).

The CREMA concept was developed to improve local community participation in natural resource management, reduce pressure on wildlife-protected areas, and improve the socio-economic development of its constituents. Significant effort has been made toward these objectives through support from both civil society organizations (CSOs) and the Wildlife Division since 2000, when the latter formalized the initiative through its Collaborative Resources Management Policy (WD 2000). However, little progress has been made to monitor the CREMA’s effectiveness or its ability to be self-sustaining. While a national review of CREMAs was completed in 2020 (Agyare et al. 2020), it was not comprehensive, with some CREMAs excluded from the assessment. For example, none of the six CREMAs of the Western Wildlife Corridor (WWC) in northern Ghana —the focus of this study—were included in the assessment.

Studies have shown that the CREMAs, including those in the WWC, face difficulties related to the effectiveness of their governance bodies, financial and technical resources, adequate inclusion of diverse interest holders, and persistent conflicts between various resource users (Agyare et al. 2015, 2020, 2024; Baruah 2017; Ahmed and Gasparatos 2020; Bayala et al. 2020; Mawutor and Hajjar 2024; Reed et al. 2024) This, therefore, raises concerns about the capacity of actors to ensure appropriate governance of these CREMAs in order to effectively reconcile biodiversity conservation and rural development.

Many authors have studied CREMAs from various perspectives, including their organization and operation (Agyare 2013; Asare et al. 2013; Murray et al. 2018), interest holders’ expectations of CREMAs and perceived benefits (Agyare et al. 2015), perceptions of governance challenges (Adeyanju et al. 2021; Asare et al. 2013; Baddianaah and Baaweh 2021; Baruah et al. 2016; Bayala 2023; Foli et al. 2018), natural resource use conflicts, changes made and future landscapes desired (Adeyanju et al. 2021; Ahmed & Gasparatos 2020; Bayala et al. 2023a; Bayala et al. 2023b). However, it is rare to find studies that investigate the capacities of CREMAs and even more so in the context of the WWC, with Agyare et al. (2020) as one of the few exceptions. This study intends to fill this gap by testing a self-assessment tool to assess the governance capacity of CREMAs in the WWC. The study aims to analyze the capacities of these CREMAs to perform their institutional roles, achieve livelihood and conservation objectives, and participate in broader multi-actor decision-making processes.

The main research question that guides the study is: How do actors assess the capacity of WWC CREMAs in northern Ghana to function and govern landscape resources? This question leads to the following sub-questions: How do WWC CREMAs currently perform in terms of landscape governance? What are the existing capacities and capacity gaps of stakeholders involved in governance? How can the functioning of CREMAs be improved?

The rest of the article is structured as follows. First, it outlines the theoretical framework, highlighting the link between the concept of capacity and landscape governance. Second, it describes the methodology adopted to carry out the study. Third, the article presents the results of the assessment of actors’ capacities in governing the CREMAs, presenting the existing capacities and capacity gaps. Fourth, it discusses the results of the study and proposes recommendations for overcoming some of the challenges revealed through this assessment. The final section concludes the article, arguing that capacity building and awareness raising are needed to address the weak capacity of the CREMA governance bodies to take ownership of the approach and implement it effectively.

## The Concept of Capacity in Landscape Governance

Landscape governance is the combination of formal, semi-formal, and informal processes of negotiation and adaptation (Endamana et al. 2010; van Oosten 2013; Kusters et al. 2018) that involve multi-scale decision-making by many networks of actors within a spatial framework (Kozar et al. 2014). Given that landscapes are continuously changing in terms of ecological structure and social interests, governance processes are therefore dynamic and iterative and result from complex consultations and decision-making between interest holders operating in different sectors and at different levels and scales (van Oosten 2013). Effectively navigating these processes requires that governance actors possess adequate capacity to enhance the performance of initiatives and the sustainability of the landscape. This study focuses on the conditions under which CREMA governance actors in the WWC find themselves in terms of capacity to govern. But what does the concept of capacity imply in the context of landscape governance?

This article refers to the conceptualization of ‘landscape governance capacity’ (by van Oosten et al. 2016, p.2) as “The collective capabilities of landscape actors to govern their shared landscape from an integrated perspective, with a view to shared concerns and goals and in connection with dynamics beyond that particular landscape.” According to this definition, supported by Arts et al. (2017), the capacities required for landscape governance can be disaggregated as in Table 1.

**Table 1:** The five types of capabilities required for landscape governance

No.	Capabilities	Implications
1	Capability to ‘think’ landscape	Being able to understand the identity, dynamics, strengths, and opportunities of the landscape and to act strategically accordingly. This means understanding the natural and ecological characteristics of the landscape, as well as its socio-cultural identity.
2	Capability to achieve internal coherence	This implies having the qualities needed to handle the diversity of actors in the landscape and the ability to facilitate collaboration between them, including leadership and the skills to stimulate multi-actor networks, establish a shared vision, leverage power relationships, and manage conflict.
3	Capability to make institutions work for landscapes	Refers to the ability to recognize and capitalize on endogenous landscape institutions, securing access rights to resources and benefits, and building new institutions connected to wider policy frames and markets.
4	Capability to create landscape market value	Being able to stimulate entrepreneurship, create sustainable business models for the landscape, and attract funding.
5	Capability to manage resources	Necessitates an in-depth understanding of resource dynamics and endogenous management systems as well as determining possibilities for resource management systems, participatory spatial planning, and decision-making that are more grounded in science.

Source: Adapted from van Oosten et al. (2016) and Arts et al. (2017)

Table 1 demonstrates that governing the landscape requires a broad range of technical and analytical skills, reflecting leadership, entrepreneurship, and inclusive management qualities. Acquiring these skills necessarily involves building the capacity of governance stakeholders, particularly local communities, through a long-term process that encompasses various training and awareness-raising initiatives aimed at creating, improving, strengthening, and transferring technical skills, know-how, and knowledge to stakeholders (Ika and Donnelly 2017; Ahmed 2022; Kacou et al. 2022).

## Methodology

### The Study Area

**Fig. 1:**
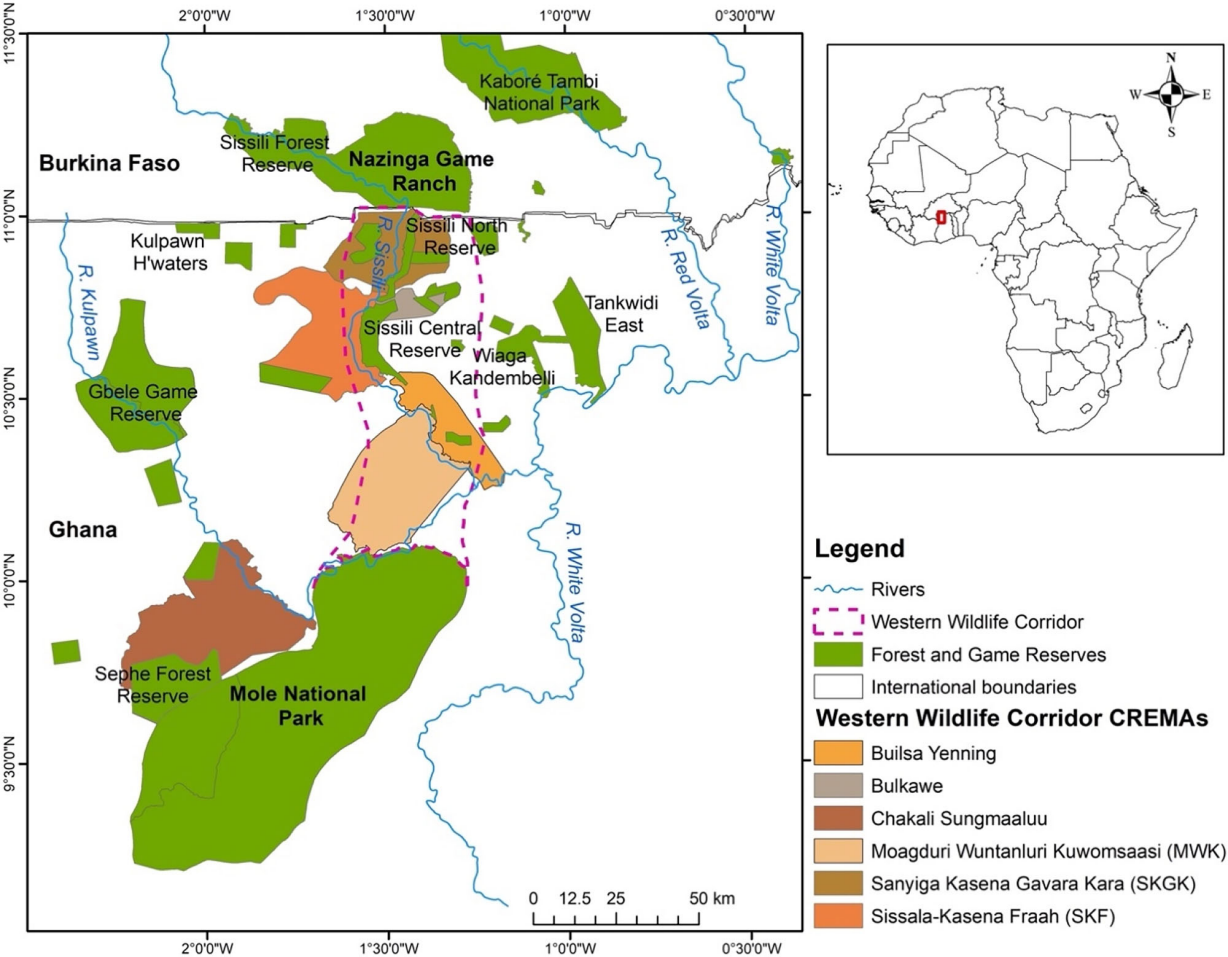
The study area. Source*:* Adapted from Ouedraogo et al. (2007) based on data from the Ghana Forestry Commission and ArcGIS hub

This study was carried out in the Western Wildlife Corridor (WWC) in Ghana, which lies in the savannah ecological zone of northern Ghana and consists largely of shea parklands (Fig. 1). It focuses on six CREMAs, namely Builsa Yenning, Bulkawe, Chakali Sungmaalu, Moagduri Wuntanluri Kuwomsaasi (MWK), Sanyinga Kasena Gavara Kara (SKGK) and Sissala Kasena Fraah. Despite intense pressure on natural resources, this landscape remains home to diverse flora and fauna that play a vital role in supporting local livelihoods (Braimoh and Vlek 2005). However, the majority of rural people in northern Ghana experience extreme poverty exacerbated by a difficult socio-economic context and high vulnerability to climate change (Abdul-Moomin et al. 2016; Bayala et al. 2020; Bayala et al. 2023b). Local communities are mainly from the following ethnic groups: Dagbamba, Sisala, Dagaba, Kasena, Bulsa, Mamprusi, Wala, Chakali, Lobi, and minority ethnic groups such as the Hausa, Fulani, and Mossi (Awedoba 2006; MoFA n.d.). These communities depend on natural resources, resulting in high pressure on natural ecosystems, leading to the degradation and fragmentation of the landscape (Barlow et al. 2018; Bayala et al. 2023b).

The primary source of income is agriculture, which is mostly rain-fed and dependent on the preservation of soil fertility (Owusu-Ansah 2018). In addition, local communities earn a living through hunting, charcoal making, mining, livestock, selling forest products, and petty trade (food processing and weaving) (Bayala et al. 2023b; Marchetta 2011). Tropical, multifunctional landscapes such as the WWC, are characterized by a mosaic of fragmented forests, areas of agroforestry and monocultures, as well as human settlements, which offer the potential to combine livelihood development and biodiversity conservation strategies (Grass et al. 2020). Thus, the WWC landscape constitutes a source of opportunities but also conflict for many actor groups, such as the local landscape users, the practitioners engaged in conservation and sustainable resource use, and private sector actors from outside the landscape, as they pursue often-conflicting claims over natural resources in support of livelihoods, conservation, and development (Bayala 2023).

This study area was selected as part of the multi-country project, “Collaborating for Operationalising Landscape Approaches for Nature, Development and Sustainability (COLANDS),^1^ led by the Center for International Forestry Research (CIFOR), which is being implemented in Ghana, Zambia, and Indonesia (Reed et al. 2020a). For this study, twelve communities were selected across the six CREMAs, two per CREMA. These communities were selected based on a categorization of the communities in each CREMA into two groups: the group of communities with very active CRMCs and the group of communities with less active CRMCs. The ‘very active’ and ‘less active’ characteristics were defined by the CREMAs’ chairpersons based on the criteria of meeting reliability, accessibility (being easy to reach), and dynamism. Thus, the CRMCs that meet these criteria are considered very active, and those that do not are considered less active. Once the CREMA chairperson established the two categories, he drew lots within each to select the community to be visited for the study. Table 2 shows the list of villages selected and visited by category and by CREMA.

**Table 2:** Communities studied by CREMA and by category

CREMA	Number of communities	Selected very active CRMCs	Selected less active CRMCs
Builsa Yenning	10	Pintengsa	Musidema
Bulkawe	15	Kandema	Yikpieng
Chakali Sungmaalu	21	Sogla	Jayiri
Moagduri Wuntanluri Kuwomsaasi (MWK)	11	Wutubri	Yikpabongu
Sanyinga Kasena Gavara Kara (SKGK)	10	Baliu	Kwapun
Sissala Kasena Fraah	23	Mwanduonu	Fachoboi

Source: Field data, 2023

### Data Collection

Data was collected using two methods: focus group discussions and key informant interviews. The focus groups were conducted with the leaders of the CRMCs of the selected communities and the CECs. A total of 18 focus groups were held involving 41 CREMA leaders. The guide used for the focus groups was built on a self-assessment tool developed for CREMAs by the Organisation for Indigenous Initiatives and Sustainability (ORGIIS-Ghana) to help them evaluate their performance over time (see Online Resource 1). It is divided into three areas of capacity as follows:

- **Governance, organizational, and program management**: this domain focuses on governance structures and guiding principles that make the CREMA work, including vision/mission setting, strategy/action plan development, decision-making and participation, information flow, transparency and accountability, conflict resolution, grievance, and redress mechanisms.
- **Operational and institutional capacities:** the focus is on skills and capacities for planning and implementing projects/activities, such as problems or needs identification, proposal writing/fundraising, reporting, and capacity for procurement and equipment maintenance.
- **Best practices on cross-cutting themes**, such as gender and social inclusion, partnerships and external cooperation, monitoring/patrolling, development of livelihood initiatives, and financial sustainability.

The self-assessment tool was designed to give each governance body (CRMC and CEC) a score reflecting its performance. The performance scores were categorized as follows:

○ Less than 30%—non-performance: the CREMA barely exists or is dormant and requires urgent action in terms of capacity building to ensure its survival.
○ Between 30 and 50%—poor performance: the CREMA is less functional but can improve with some level of technical assistance.
○ Between 51 and 70%—good performance: the CREMA is functioning successfully, shows a relatively high level of performance, and can be maintained with minimal technical assistance.
○ Above 70%—strong performance: the CREMA is robust, effective, and self-sustaining.

Key informant interviews were conducted with community opinion leaders, including traditional chiefs, queen mothers,^2^ religious leaders, heads of local cooperatives, local people, Fulani herders, and institutional actors such as officers of the Wildlife Division and Forest Services Division (FSD) of the Ghana Forestry Commission, and District Assemblies. In each of the target communities, apart from the opinion leaders available to take part in the study, three residents—one woman, one man, and one male or female youth who were not members of the CRMC—were randomly selected for an interview. In three localities where they could be found, a Fulani^3^ man was interviewed. A total of 53 individual interviews were conducted from March to April 2023: 44 in the 12 target communities and nine with policymakers, using interview guides (see Online Resources 2 and 3).

Participation in the interviews and focus groups was voluntary. Direct quotes are used to support findings. However, for ethical reasons, we kept the identities of the participants in the study confidential and do not provide information that might allow them to be identified. Data collection took place from March to April 2023. Table 3 shows the number of focus groups and individual interviews carried out by locality.

**Table 3:** Overview of the focus groups and individual interviews conducted per CREMA

Data collection activity	Locality	Stakeholders interviewed	Number of focus groups/interviews
Focus group discussions	Builsa Yenning	CEC of Builsa Yenning	1
		CRMC of Pintengsa	1
		CRMC of Musidema	1
	Bulkawe	CEC of Bulkawe	1
		CRMC of Kandema	1
		CRMC of Yikpieng	1
	Chakali Sungmaalu	CEC of Chakali Sungmaalu	1
		CRMC of Sogla	1
		CRMC of Jayiri	1
	Moagduri Wuntanluri Kuwomsaasi (MWK)	CEC of MWK	1
		CRMC of Yikpabongu	1
		CRMC of Wutubri	1
	Sanyinga Kasena Gavara Kara (SKGK)	CEC of SKGK	1
		CRMC of Kwapun	1
		CRMC of Baliu	1
	Sissala Kasena Fraah	CEC of Sissala Kasena Fraah	1
		CRMC of Fachoboi	1
		CRMC of Mwanduonu	1
	TOTAL		18
Individual interview	**Communities (44)**		
	Wutubri	Women (1), Men (1), Youth (1), TA (1), Landlord (0), Fulani (0)	4
	Yikpabongo	Women (1), Men (1), Youth (1), TA (1), Landlord (), Fulani (1)	5
	Kandema	Women (1), Men (1), Youth (0), TA (0), Landlord (0), Fulani (0)	2
	Yikpieng	Women (1), Men (1), Youth (0), TA (1), Landlord (0), Fulani (0)	3
	Sogla	Women (1), Men (0), Youth (1), TA (0), Landlord (1), Fulani (0)	3
	Jayiri	Women (1), Men (1), Youth (1), TA (0), Landlord (1), Fulani (1)	5
	Baliu	Women (0), Men (0), Youth (1), TA (0), Landlord (0), Fulani (0)	1
	Kwapun	Women (2), Men (0), Youth (1), TA (1), Landlord (0), Fulani (0)	4
	Fachoboi	Women (1), Men (1), Youth (1), TA (1), Landlord (0), Fulani (0)	4
	Mwanduonu	Women (1), Men (1), Youth (1), TA (1), Landlord (0), Fulani (1)	5
	Musidema	Women (1), Men (1), Youth (1), TA (1), Landlord (0), Fulani (0)	4
	Pintengsa	Women (1), Men (1), Youth (1), TA (1), Landlord (0), Fulani (0)	4
	**Institutional actors (9)**		
	Forest Services Division	Tumu, Navrongo, Chakali	3
	Wildlife Division	Gbele game reserve, Bolgatanga	2
	District assemblies	Builsa north, Mamprugu Moagduri, Tumu, Kasena Nankana	4
	TOTAL		53

Key: CEC = Community Executive Committee; CRMC = Community Resource Management Committee; TA = Traditional Authority (Chief or Elder). Source: Field data, 2023

### Data Processing

The variables evaluated focused essentially on the organization of CREMA governance, the skills and capacities of local governance actors, and natural resource management practices, all of which helped to measure the performance of the management bodies. Each of the variables was assessed based on indicators with evaluation scores (see Online Resource 1). These quantitative self-assessment data from the CREMAs was processed and analyzed using MS Excel spreadsheets to weigh the scores attributed to the CRMCs and CECs and to obtain an average score per CREMA. The qualitative data was transcribed in MS Word files. A thematic analysis was then carried out, consisting of a careful examination of the information gathered from the interviews and focus groups to identify common themes. The following themes were identified: the current performance of the CREMA, existing capacities, gaps in local and institutional actors’ governance capacity, and recommendations for better outcomes from CREMAs in terms of conservation and livelihoods.

## Results

### CREMA Performance Self-Assessment

Table 4 presents the results of the CREMA self-assessment completed by the leaders of the CECs and CRMCs studied. The results reveal one strong performance (above 70%), nine good performances (above 50%), six poor performances (30–50%), and two non-performance (below 30%) among the 18 CREMA governance bodies visited.

**Table 4:** CREMAs performance assessment

CREMAs	Governance bodies	Score per governance body	Score in %	Performance Index
Builsa Yenning	CEC of Builsa Yenning	59/111	53%	52%
	CRMC of Pintengsa	53/111	48%	
	CRMC of Musidema	61/111	55%	
Bulkawe	CEC of Bulkawe	67/111	60%	58%
	CRMC of Kandema	61/111	55%	
	CRMC of Yikpieng	65/111	59%	
Chakali Sungmaalu	CEC of Chakali Sungmaalu	40/111	36%	40%
	CRMC of Sogla	38/111	34%	
	CRMC of Jayiri	54/111	49%	
MWK	CEC of MWK	52/111	47%	42%
	CRMC of Yikpabongu	63/111	57%	
	CRMC of Wutubri	24/111	22%	
SKGK	CEC of SKGK	90/111	81%	62%
	CRMC of Kwapun	57/111	51%	
	CRMC of Baliu	61/111	55%	
Sissala Kasena Fraah (SKF)	CEC of Sissala Kasena Fraah	57/111	51%	37%
	CRMC of Fachoboi	16/111	15%	
	CRMC of Mwanduonu	49/111	44%	

The number 111 refers to the cumulative maximal score of all indicators assessed

Source: Field data/ FGDs, 2023

The cumulative performance scores of each of the six CREMAs indicate the following two categories of CREMAs:

- Less functional CREMAs in the poor performance class (30–50%) that can improve with some level of technical assistance: Chakali Sungmaalu, MWK, and Sissala Kasena Fraah.
- CREMAs in the good performance class (51–70%) that function relatively successfully with minimal technical assistance: Builsa Yenning, Bulkawe, and SKGK (but see the section on methodological reflection concerning biases in self-scoring).

The results point to a need to strengthen the capacities of some of the CREMAs to reinforce the governance of the WWC landscape to meet social and conservation needs. The sections below describe the strengths and weaknesses of the CREMAs in terms of their capacity to effectively perform landscape governance. In most cases, the level of activity made little difference in the self-assessment scores at the CMRC level: regardless of being active/less active, the self-assessments were similar, and in one case—the MWK CREMA—the less active scored significantly higher, although the reverse was the case for SKF. The rest were reasonably similar.

### Existing Capacity and Opportunities for Implementing the CREMA Initiative

Some specific capacity-building actions were taken in three CREMAs before they were established (MWK, Bulkawe, SKGK). These relate to occasional training and awareness-raising sessions provided by projects and NGOs on issues relating to uncontrolled bushfires, logging, the conservation of natural resources, and the CREMA initiative. Although carried out a long time ago—nearly ten years, according to the interviewees—these actions have helped to improve the capacity of many local people, enabling them to better understand environmental issues and to be more receptive to conservation initiatives.

Some CREMAs, notably Bulkawe, SKGK, and Builsa Yenning, have benefited from support from ORGIIS-Ghana, an NGO, to set up a team to conduct regular ecosystem monitoring and surveillance patrols. The existence of the CECs and CRMCs, which are multistakeholder platforms at the community and landscape levels, is already an asset in terms of operational capacity for the governance of the CREMAs and the management of conflicts relating to the use of natural resources. The focus groups show that the CREMA governance bodies, as well as the chiefs’ palaces, are the local courts where conflicts are managed.

All the local CREMA leaders interviewed stated that they have the skills to lead their CREMA, mobilize communities and facilitate events, speak up and advocate for community members, raise awareness on issues, establish and manage tree nurseries, conduct patrols and report violations, monitor and report on activities, and keep good records. These skills need to be put into practice, but the CREMAs have neither the financial nor the material resources to do so, they argued.

The state agencies (Wildlife Division, FSD, District Assemblies) have the technical capacity, equipment, and—some of them—means of transport to support the governance of the CREMAs. However, they lack the financial support needed to provide concrete support to the CREMAs. Furthermore, the private sector actors and NGOs present in the WWC landscape (Noé, ORGIIS-Ghana, CIFOR, the Savanah Fruits Company, the Ghana Shea Landscape Emission Reduction Project) represent an opportunity to be seized to strengthen the capacities of the CREMAs and improve their functionality. To achieve this, lobbying and partnership negotiations would be useful to optimize collaboration between these interest holders and the CREMAs.

The findings reveal a commitment among some community members to support the governance bodies by alerting them in the event of illegal activities in the CREMAs. Likewise, the commitment of the traditional chiefs to support the CREMA initiative was well evidenced: all the traditional authorities interviewed were in favor of the CREMAs, suggesting, moreover, that they should function better. This enthusiasm for the CREMAs, however limited, is an asset that should be capitalized upon to enable and strengthen the participatory monitoring of the resources of the WWC landscape. Unfortunately, this study also revealed capacity gaps, which will be discussed in the next subsections for communities, CREMA governance committees, and institutional actors, respectively.

### Actors’ Capacity Gaps in CREMA Governance

#### Local Communities

Interviews with local communities in the CREMAs revealed gaps in their understanding of the CREMA concept. Indeed, most interviewees (82%) claimed that they did not understand the concept well. Those (18%) who stated that they had good knowledge of it acquired their knowledge through participation in meetings organized during the implementation phase of the CREMAs or through other people and defined it simply as an area where logging, poaching, and grazing are strictly prohibited. However, they do not see the CREMA concept as an approach to landscape governance in which they are at the center of decision-making and through which they should attempt to advance rural development while also sustainably managing resources. On the contrary, they see it as a set of restrictions, privations, and prohibitions on the use of natural resources. Basically, these communities are unfamiliar with the policy documents governing the CREMAs and, consequently, with the rules applicable to the use of natural resources. This situation also applies to most members of the CRMCs who hold positions within the governance body but have only a vague knowledge of the implications of the CREMA concept and do not know how to perform their role fully in the governance process.

In all the communities visited, the interviewees said that they were willing to support CREMA actions if they were informed and involved. However, they lamented the lack of capacity-building opportunities to enable them to understand better and engage more fully in the CREMA governance system, with some participants stating:

“We lack opportunities to get trained, yet there is a need for capacity building to update committee members and inform people so that they engage more in CREMA work” (TA1-MWK).

“I would like to learn more about the CREMA issues, and it will be beneficial for the community to also know more about CREMA because they cannot help properly if they don’t understand” (Woman 2-Mwanduonu/SKF).

**Fig. 2:**
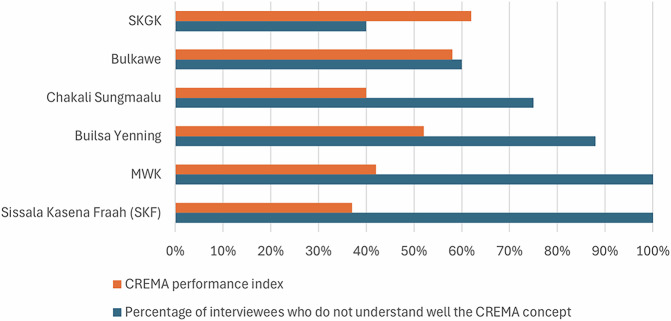
Correlation between CREMA performance and knowledge of the CREMA concept. Source: Field data/individual interviews, 2023

The study shows that communities’ lack of knowledge of the CREMA concept influences the proper functioning of CREMAs. Figure 2 presents a correlation between the CREMA performance index and the number of people who do not understand the CREMA concept well.

Figure 2 shows that CREMAs with a higher percentage of people who do not fully understand the CREMA concept generally perform worse than those with a lower percentage of such individuals. However, the Chakali Sungmaalu CREMA is an exception to this trend in that it has a low performance index (40%) compared with Builsa Yenning (52%) and MWK (42%), which nevertheless have higher rates of people who do not understand well the CREMA concept (Chakali Sungmaalu = 75%; Builsa Yenning = 88%; MWK = 100%).

#### CREMA Governance Committees

As outlined in the introduction, two governance bodies support natural resource use planning, democratic decision-making, community-based management, and benefit sharing for all CREMA stakeholders: the CRMC and the CEC. The research findings show that the CREMAs in the WWC face a number of constraints that prevent the governing bodies from functioning properly and ensuring adequate governance to achieve the desired objectives. The most important of these are related to deficient equipment and financial means, a lack of inclusiveness in CREMA governance, and the lack of partnerships and collaborations between CREMA governance bodies and other institutional actors and organizations.

- *Deficient equipment and financial means*
  With the exception of MWK CEC, which has an office building in Yizesi, none of the other WWC CREMAs has one. Even the MWK CEC office remains unused due to a lack of equipment. Only the CEC of SKGK has some basic office equipment, such as a computer, printer, and GPS; the other CREMAs have no such equipment. The lack of financial resources was mentioned by all the governing bodies interviewed as one of the biggest challenges facing the CREMAs. Consequently, the CRMCs and CECs are unable to organize meetings, patrols, or other socio-economic activities and to mobilize communities around the initiative. The inability of these governance bodies to organize meetings is said to be the reason why many of their officers do not serve for more than one term after their terms in office have expired. The CREMA local leaders work on a voluntary basis, and it appears that many of them are demotivated by the lack of financial or material benefits. As a result, they are no longer fully committed to the success of the initiative. One of the difficulties in terms of financial resources is also the lack of local skills for formulating projects and seeking funding from accredited institutions. The inability to initiate self-financing initiatives is also a weakness that makes the CREMA governance bodies dependent on external (limited) funding. The evaluation also revealed a lack of conditions enabling local CREMA leaders to ensure sound financial management. The leaders interviewed admitted that they did not have the necessary skills in the finance domain, and of the 18 governance bodies assessed, only the Bulkawe CECs had a bank account.
  According to the interviewees, the lack of financial resources to initiate social projects to improve and diversify livelihoods is a significant handicap that does not motivate them to engage. They fail to see the impact of CREMAs in terms of improving their living conditions and, rather, only feel prohibitions. For these people, initiatives are needed to mark the existence of CREMAs. Most of them mentioned setting up home gardens in the communities, creating nurseries for plant production, installing dams or boreholes to facilitate dry-season gardening, and developing alternative livelihood options such as carpentry, welding, trade, and animal husbandry.^4^ In terms of support for the socio-economic development of vulnerable groups, only the Builsa Yenning CEC reported having benefited from a grain mill for women. However, this equipment was no longer operational during the period covered by this study because of a breakdown and lack of funds for maintenance.
  In addition, the governance bodies do not have sufficient equipment to enable them to carry out ecological monitoring of the landscapes. The CECs of all six CREMAs stated that they only had either a tricycle^5^ or a motorbike, which in most cases are not in working order, or working boots and a machete. Furthermore, none of the CREMAs has a landscape development and management plan, either at the CRMC level or CEC level, representing a clear handicap for the governance of natural resources.
- *Poor CREMA governance and limited inclusivity*
  According to the participants in the FGDs with CREMA leaders, CEC and CRMC members’ knowledge of natural resource governance is limited and needs to be improved. The issue of inclusion is considered in the texts that frame the CREMAs (constitutions, CREMA policy) (Asare et al. 2013), but in practice, the results of this study revealed that inclusive governance is still far from being achieved in WWC CREMAs. Most CRMCs and CECs have a disproportionate representation of men in committees (made up of between 9 and 14 members) compared to women (between 1 and 3). The same applies to the Fulani ethnic group, who were only represented in 2 out of 6 CECs and 4 out of 12 CRMCs. In this specific case, which is particularly sensitive due to deep-rooted historical and ongoing conflicts, hostility is often openly expressed:
  “There is no Fulani in the committee because they are not qualified. They don’t have the competencies, there is a language barrier, and they are not well known” (FGD-7 Bulkawe/25-03-2023).
  “We sacked the Fulani; we don’t need them on our land” (FGD-10 SKGK/28-03-2023).
  “We prefer not to include the Fulani. Communities don’t want them to be part” (FGD-13 SKF/30-03-2023).
  In Fachoboi in the Sissala Kasena Fraah CREMA, there was no formal CRMC at the time of this study. A small group of five people (all men) had voluntarily come together to look after the management of their community’s natural resources. None of the four people interviewed and four FGD participants in this community had heard of the CREMA initiative.
- *A lack of partnerships and collaborations*

The results of this assessment showed that none of the CREMAs visited had a formal partnership with an organization for the sale of local agricultural products or non-timber forest products. These products are generally sold on local markets, usually individually and very rarely collectively, for instance, through cooperatives.

It emerged from the interviews that collaboration between the CREMAs and external stakeholders is generally tacit, without formal agreements. With certain relevant state agencies in the landscape (i.e., the Forest Services Division), this collaboration is deficient due to institutional conflicts of interest. Six CREMA leaders and one traditional authority mentioned the existence of tension between the FSD and the Wildlife Division of the Ghana Forestry Commission concerning the governance of the CREMAs. According to them, FSD officers do not pay attention to the CREMAs because these fall within the remit of the Wildlife Division. Yet this department also has a major role to play in conserving forest resources and preserving ecosystem services within the CREMAs. According to one of the traditional leaders:

“When forest officers (FSD) come to work in communities, they don’t involve the CREMA committee members; it’s not good” (TA 1-SKF).

In addition, local CREMA leaders in the FGDs mentioned the lack of advocacy and relationship-building skills needed to develop their CREMAs as a key barrier to effective landscape governance.

#### Institutional Actors

The main state institutions responsible for supporting CREMA functioning— the Wildlife Division and District assemblies—lack the resources to fulfill their role. According to these actors, the lack of staff, financial resources, and—in the case of the District Assemblies—the means of transport are the main limitations that prevent them from supporting the CREMAs in functioning optimally. As they do not benefit from state budget allocations for the CREMA operations, they are limited essentially to carrying out basic activities, such as surveillance, control, and protection of natural resources, and rely on possible support from projects and NGOs to carry out more substantial actions in favor of better development of the CREMAs. According to an institutional actor interviewed:

“The concept of CREMA is good, but it needs to be reinforced. It should consider more of the human aspect, not only wildlife, and for that, more financial capacities are needed to implement projects” (Forest Officer FC/FSD).

Six of the nine institutional actors interviewed—mainly District Assembly members—stated that they did not have a solid knowledge of the CREMA concept, thus highlighting the need for training to enable them to fully understand their role in providing appropriate support. In addition, most of these actors—particularly the District Assemblies and FSD—noted the very weak or even lack of collaboration between them and the local CREMA governance bodies. Weak inter-institutional collaboration was also noted, especially between the Wildlife Division and FSD (see also the previous section). Interviews with these institutional players revealed that within the Forestry Commission, which is the national institution in charge of natural resource governance, the promotion and administrative management of CREMAs is the exclusive jurisdiction of the Wildlife Division. The FSD does not feel involved, and this has created a rift in collaboration between the two departments on the issue of CREMAs.

## Discussion

### Landscape Governance Requires Appropriate Capacities

This study emphasizes the need to build capacity in community-based natural resource management approaches, specifically within Ghana’s CREMA model. The findings highlight various obstacles to the effective functioning of the CREMAs in the WWC, largely related to the weak capacity of the governance bodies to take ownership of the approach and implement it effectively. Thus, actors’ commitment alone is not enough. In fact, the results show that the level of commitment of the CREMAs’ actors does not necessarily reflect their performance; some CRMCs deemed to be less active had higher performance scores than those considered to be very active. Current capacities do not allow local governance actors to initiate a participatory and inclusive multi-actor process. This could be attributed to limited awareness and knowledge of the CREMA concept, limited funds to mobilize actors and implement activities or support CREMAs, and a lack of partnerships and inter-institutional collaborations, particularly with organizations operating beyond the community level.

CECs and CRMCs constitute community-level and landscape-level multi-actor platforms but are neither sufficiently inclusive nor fully functional and are highly dependent on external funding. Authors such as Agyare et al. (2015, 2020), Asare et al. (2013), and Bayala et al. (2020, 2024) have made similar observations in their studies on CREMAs in different areas. Yet, improved functioning of these platforms could facilitate better communication among interest holders on landscape challenges and identification of lessons learned, shared concerns, and opportunities for collaboration (Bayala 2024). Furthermore, the current state of knowledge of local actors does not allow them to either lead or meaningfully contribute toward such multi-actor processes. According to Agyare (2013), CREMA governance committees do not have sufficient abilities to manage group discussions and, less so, cross-cultural groups, which is consistent with the findings of this study. This poses a barrier to sustainable landscape governance.

In their current state of operation, it is difficult for the WWC’s CREMAs to fully meet the objectives of improving the living conditions of populations and conserving biological diversity in the landscapes. The study showed that the majority of interviewed people in the CREMAs, including the leaders of the governance bodies, do not sufficiently understand the CREMA concept, representing a significant handicap to their active involvement in the management mechanisms and decision-making processes and an obstacle to the application of inclusive and iterative governance. Communities need to have an adequate understanding of the approach being implemented in their area, which requires open collaboration and local ownership. Indeed, CREMA’s performance appears to be closely linked to the number of people who understand the CREMA concept. The study reveals that the higher the percentage of people who understand the concept, the better the performance. A proper understanding of the CREMA concept by the communities could have a positive impact on the functioning and performance of the CREMAs.

Furthermore, the results reveal a classic case of devolution of authority by name only. The government has devolved authority to the local level to manage the natural resources but failed to provide adequate technical, financial, material, and livelihood support, as well as the power to enforce CREMA regulations. Therefore, communities that lack resources, knowledge, and power to implement well-meant objectives remain incapacitated while facing the increased burden of responsibility to sustainably manage and conserve resources. A national assessment of CREMA performance, focusing on eight CREMAs in different regions of the country, highlighted the lack of adequate support from the state as a barrier to optimal performance (Agyare et al. 2020). In terms of leadership, the results of the study indicate an inability of local CREMA actors—both community and state actors—to mobilize interest holders to enact a multi-actor dialog, initiate resource mobilization, or develop partnerships for the betterment of the CREMAs. According to Agyare et al. (2020), strong and effective leadership, as well as good team spirit, are essential qualities for managing CREMAs effectively, yet they are often lacking in certain regions, as is the case in the WWC.

The results also show that significant emphasis is placed on the environmental dimension rather than the socio-economic dimension, particularly regarding developing existing governance capacities. The support and training received previously were more oriented toward achieving conservation objectives. Hence, community actors lament the limited or lack of livelihood development opportunities and interventions. For a CREMA to function effectively and be better accepted by local communities, training these communities in the sustainable use of forest and wildlife resources is crucial so that they have the necessary skills and knowledge to use biodiversity conservation as a means of sustaining their livelihood (Agyare et al. 2020). Such capacity building should form a fundamental component following the implementation of CREMAs, whereas creating livelihood benefits is necessary for the buy-in of the CREMA concept in the communities. Currently, community members perceive the CREMA as more of a restriction than a mechanism that can help improve their livelihoods and increase their voice in natural resource governance.

Despite the significant constraints, some CREMA leaders consider their performance to a reasonable standard, as evidenced by the high scores in the self-assessment. Unfortunately, this may not necessarily reflect the actual performance of the CREMAs due to potential self-assessment biases (see Methodological Reflection section). However, the fact that CREMA executives remain enthusiastic and willing to continue their engagement points to a potential leverage point for improving the future performance of CREMAs in the WWC. However, this remains contingent on the provision of adequate support from external actors (Bayala 2024). This study proposes that most of this support should come from the government, given that it initiated the CREMA concept and the Wildlife Division is the primary entity responsible for the maintenance of CREMAs.

Community-based natural resource management approaches are often criticized for underperforming in achieving conservation and livelihood goals, contributing only marginally to household well-being, as observed in Botswana and Tanzania (Pailler et al. 2015; Cassidy et al. 2023). However, assessing these initiatives can be challenging, as they are designed to fulfill a range of environmental and socio-economic objectives. Consequently, their success or failure depends on the specific outcomes considered (Mawutor and Hajjar 2024). This study shows that the capacities of governance actors are a key element that should be prioritized in community-based approaches. To enhance the potential to effectively reconcile the conservation of biological diversity and local development, particularly in the context of community-based approaches, it is necessary to ensure that local communities possess the required capacities to conduct the processes optimally. This involves initiating awareness-raising, training, and workshops to discuss and share appropriate knowledge to address needs and challenges, as well as providing technical assistance to local stakeholders to gradually empower them (Ika and Donnelly 2017; Kacou et al. 2022). In the same vein, Morkel and Ramasobama (2017) argue that in Africa, capacity-building initiatives are most often based on the training of individuals backed by technical support. This is equally applicable to various approaches with similar objectives, including approaches recognized in the literature as currently holding potential and generating donor and policy support, such as REDD+ and integrated landscape approaches (ILAs) (Luintel et al. 2013; Reed et al. 2020b). The ILA principles, for instance, specifically emphasize capacity building for actors (Principle 10) and continuous learning (Principle 1) (Sayer et al. 2013) to ensure that actors involved in landscape governance processes are well-resourced and updated. Capacity building is multidimensional, dynamic, and complex, influenced by factors such as people’s existing capacities, knowledge, behaviors, and attitudes, as well as the context in which the individual or community is situated (Morkel and Ramasobama 2017). Learning from the implementation of capacity-building initiatives in Vietnam, Sri Lanka, Indonesia and Ghana, Ika and Donnelly (2017) argue that strong stakeholder engagement, open collaboration between implementing actors, alignment with context priorities, and adaptation or flexibility are necessary conditions for successful capacity-building initiatives.

### Implications and Recommendations

The results of this assessment of CREMA governance capacities in the WWC helped identify some key avenues toward enhancing the functioning of CREMAs and their potential for improved performance and outcomes.

First, to improve landscape governance, the study highlighted the necessity for the Wildlife Division as the main promotor of CREMAs to invest in the capacities of governance actors, especially through training of CEC and CRMC members in the CREMA concept and design, the constitutions and by-laws governing the functioning of CREMAs, and fundraising, as well as in key concepts such as inclusive governance, sustainable development, and related processes. Interviews with governance actors revealed the need for support in setting up and strengthening the capacities of patrol teams for ecological monitoring in CREMAs.

Pending support from external partners/donors, the Government of Ghana, as the initiator of the CREMA system, should commit itself more fully to ensuring the success of the CREMA initiative by allocating annual budgets to relevant institutions to enable the CREMAs to operate at least minimally. This would make it possible to meet basic needs such as office space for CECs, office supplies, computers, desks, and chairs; means of transport; personal protective equipment such as gloves, boots, and cutters; funds for training, sensitization and awareness-raising initiatives for natural resource management and biodiversity conservation in CREMA communities. Moreover, it is recommended that a participatory monitoring system be established under close supervision by officials from the Wildlife Division of the Ghana Forestry Commission.

Better technical and financial capacities would enable regular meetings between CREMA leaders and community members to document activities and discuss the progress and challenges in landscape management, support the production of development and management plans at community and landscape levels, and support monitoring that feeds processes of adaptive management. To enhance the sustainability of CREMAs, local governance bodies should be supported to develop self-financing strategies, for instance, through improved access to relevant finance and markets, systems for payments for ecosystem services (PES), valuing traditional knowledge, eco-tourism, improved project design, and fundraising actions (Bayala 2024).

The study also highlighted a lack of collaboration between the two main entities responsible for forest/landscape governance in Ghana, despite increasing evidence of good collaboration between interest holders being one of the key factors in the success of landscape-scale natural resource and conservation initiatives (Deans et al. 2018; Elbakidze et al. 2010; Favretto et al. 2021; García-Martín et al. 2016; Ros-Tonen et al. 2015). Open collaboration between the two entities, the FSD and Wildlife Division, is therefore considered crucial to boosting the performance of the CREMAs.

Second, it is necessary to address the lack of livelihood development interventions, which presents an obvious obstacle to the motivation of community stakeholders for meaningful engagement in the CREMA system. Introducing or strengthening sustainable livelihood options would, therefore, be a pathway toward greater support for the CREMA approach. Discussions with the WWC governance actors generated several recommendations on ways to enhance sustainable livelihoods for the benefit of local communities, including the development of value chains based on non-timber forest products such as shea (*Vitellaria paradoxa*), dawadawa (*Parkia biglobosa*), and baobab (*Adansonia digitata*), fostering off- or dry season farming, and capacity building in climate-resilient agro-silvopastoral practices.

Furthermore, according to interviewees and FGD respondents, incentives for developing livelihood options that provide alternatives to farming and logging should be considered to improve both well-being and natural resource conservation. However, evidence demonstrating that alternative livelihood interventions generate significant and sustainable conservation and livelihood benefits remains scarce (Roe et al. 2015). Successful alternative livelihood initiatives are typically donor-funded and often collapse once funding ends (Hilson and Banchirigah 2009; Wicander and Coad 2015; STAP 2024). This aligns with Adeel and Safriel’s (2008) argument that building communities’ technical and managerial capacity is essential for sustaining these alternative livelihoods, which they describe as ‘new businesses’ (Adeel and Safriel 2008, p138).

### Methodological Reflection

The tool used to collect the data is relevant to several specific aspects of CREMA governance. However, the fact that it is a self-assessment implies a possible bias. It is quite easy for actors in the governance bodies (CRMC and CEC) to withhold information so as not to assess themselves negatively. In an attempt to address this bias, we included key informant interviews with opinion leaders and people who were not directly involved in the governance processes to triangulate the responses obtained. However, the potential for introduced bias remains and may have influenced the final results.

## Conclusion

Landscape governance is a dynamic process involving consultation and consensual decision-making between diverse categories of actors operating at different levels and scales. Therefore, governance actors must be sufficiently resourced in terms of their ability to negotiate, design, and operationalize the proposals for land use and landscape resources. As in the case of the CREMA initiative, local communities are often entrusted with socio-economic and environmental development responsibilities without being adequately equipped to carry them out. However, the success of community-based natural resource governance initiatives is closely tied to local stakeholders’ ability to take ownership and implement them.

The results of this study show the challenges that the governance of the WWC’s landscape is facing; these are related to the lack of technical capacities of local governance actors, financial constraints, weak livelihood interventions, and lack of power to enforce CREMA rules. The study highlights the need for sound technical and financial capacity of local CREMA management actors and a strong collaboration between all key interest holders as essential factors to ensure the sustainability of CREMA governance and facilitate the integration of both conservation objectives and livelihoods. Capacity-building and awareness-raising actions for local people and governance bodies are needed to improve knowledge and understanding of the CREMA approach. Furthermore, initiatives to improve the livelihoods of local communities will be an asset in mobilizing local social groups around the CREMA approach. However, to complement this study, it would be useful to further examine how capacity-building initiatives can effectively facilitate the adoption of tools and concepts that enhance governance.

## Supplementary information

Supplementary material 1

Supplementary material 2

Supplementary material 3

## Data Availability

No datasets were generated or analysed during the current study.

## Footnotes

1 The Collaborating to Operationalise Landscape Approaches for Nature, Development and Sustainability (COLANDS) initiative is a CIFOR-led project with the University of British Columbia, University of Amsterdam, CIRAD and several local partners in the countries of implementation—Ghana, Zambia, and Indonesia— that seeks to initiate, analyse and evaluate the implementation of integrated landscape approaches. For more information see https://www.cifor-icraf.org/colands/.

2 In Ghana, Queen mothers are traditional leaders from the royal lineage, who play a strategic role in the chieftaincy set-up process, and are generally assigned responsibility for issues relating to women and children (Goldsmith et al., 2021; Owusu-Mensah et al., 2015).

3 Fulani, most of whom are nomadic pastoralists, are considered foreigners in Ghana (Tonah 2003; Bukari and Schareika 2015).

4 These are mainly small ruminants and poultry, which are reared as income-generating activities.

5 Three-wheeled motorbike fitted with a trolley, generally used for transporting materials, such as gravel, stones, and sand, agricultural products, or people in rural areas.
