# Supplementary material for: Assessing CREMAs’ Capacity to Govern Landscape Resources in the Western Wildlife Corridor of Northern Ghana
Source: Environ Manage. 2025 Apr 4;75(5):1055–70. doi: 10.1007/s00267-025-02155-9 (PMC12033088; doi:10.1007/s00267-025-02155-9)
Supplement: Supplementary file 1 — Supplementary material 1 [file 267_2025_2155_MOESM1_ESM.docx]

**Supplementary material 1**

**CREMAs’ capacity gap assessment in the Western Wildlife Corridor (WWC)**

**QUESTIONS SPECIFIC TO CREMA COMMITTEES (FOCUS GROUP DISCUSSION)**

1. **Identification**

| **Date of the assessment** |  |
| --- | --- |
| **Name of CREMA** |  |
| **Number of years in operation** |  |
| **FGD (CRMC)** | Community: |
| **Individual respondent (CEC)** | Name: Gender [ ] |
|  | Position: |
| **Name(s) of facilitator(s)** |  |

1. **Governance, organizational and program management**

| **#** | **Questions and response modalities** | **Score** | **Facilitator’s Notes/ Required Actions** |
| --- | --- | --- | --- |
| 1 | **Which statement best describes the situation of the CREMA and the level of awareness by its members?**  0 = The CREMA does not have clearly defined goals, mission, and objective statements  1 = The CREMA has clearly defined goals, mission, and objective statement, but **none of the members, including CEC and CRMC,** know about it  2 = The CREMA has clearly defined goals, mission and objective statement known by **only the CRMCs and CEC**  3 = The CREMA has clearly defined goals, mission and objective statement known by a **few of its members, including CRMCs and CEC**  4 = The CREMA has clearly defined goals, mission and objective statement known by the **majority of its members, including CRMCs and CEC**  5 = The CREMA has clearly defined goals, mission and objective statement known by **all its members**, and they can clearly articulate it |  |  |
| 2 | **Which statement best describes the CREMA governance structures set-up?**  0 = The CREMA does not have any of its basic structures (CRMCs) in place  1 = The CREMA has interim CRMCs in place  2 = The CREMA has elected CRMCs, but no CEC is in place  3 = The CREMA has elected CRMCs and CEC in place but not inaugurated  4 = The CREMA has elected CRMCs and CECs inaugurated but not fully functioning  5 = The CREMA has elected CRMCs and CECs inaugurated and fully functioning^^[[1]](#footnote-2)^^ |  |  |
| 3 | **Which statement best describes the existence and awareness of the CREMA’s constitution**  0 = The CREMA does not have a written Constitution  1 = The CREMA has a written constitution; none of the members knows its provision  2 = The CREMA has a written constitution; its provisions are known by only the CRMCs and CEC  3 = The CREMA has a written constitution; its provisions are known by a few of its members, including the CRMCs and CEC  4 = The CREMA has a written constitution; its provisions are known by the majority of its members, including the CRMCs and CEC  5 = The CREMA has a written constitution; its provisions are known by all the members |  |  |
| 4 | **Which statement best describes the state of the CREMA at the level of the District Assembly?**  0 = The CREMA’s constitution is not submitted to the District Assembly  1 = The CREMA’s constitution is approved by the District Assembly, but no District Assembly by-law backing it  2 = The CREMA’s by-law is submitted to the District Assembly but not approved  3 = The CREMA’s by-law is approved by the District Assembly but not gazetted  4 = The CREMA’s by-law is approved by the district assembly and gazetted |  |  |
| 5 | **Which statement best describes CREMA’s awareness of the devolution certificate and the right or power it gives?**  0 = The CREMA has no devolution certificate; no member is aware of rights it gives  1 = The CREMA has no devolution, but members are aware of their rights as CREMA members  2 = The CREMA has a devolution certificate; only the CRMCs and CEC are aware of the rights it gives  3 = The CREMA has a devolution certificate; few members, including CRMCs and CEC, are aware of the rights it gives  4 = The CREMA has a devolution certificate; the majority of members, including CRMCs and CEC, are aware of the rights it gives  5 = The CREMA has a devolution certificate; all the members, including CRMCs and CEC, are aware of the rights it gives and are capable of demanding those rights |  |  |
| 6 | **Which statement best describes the existence and implementation of the CREMA management plan?**  0 = The CREMA has no action or management plan in place  1 = The CREMA has a CRMC-level action plan in place but is not being implemented  2 = The CREMA has a CRMC-level action plan in place and is being implemented  3 = The CREMA has an overall management plan but is not being implemented  4 = The CREMA has an overall management plan from which annual action plans are drawn each year with clear progress markers and implemented  5 = The CREMA has an overall management plan from which annual action plans are drawn each year with clear progress markers and yearly goals are achieved |  |  |
| 7 | **Which statement best describes how decisions are made within the CREMA?**  0 = No notice is given before major CREMA meetings are held, and members are not involved in planning  1 = Prior notice is given, but members are not involved in the planning  2 = Prior notice is given; members are consulted before any major decision is taken, but their views are not taken to a small extent  3 = Prior notice is given; members are consulted, and their views are adequately taken on board to a large extent |  |  |
| 8 | **The CREMA renews the mandate of the executives after their tenure expires, as stipulated by the constitution**  0 = No  1 = Don’t know  2 = Yes |  |  |
| 9 | **The CREMA has a system to change executives when they do not perform their functions.**  0 = No  1 = Don’t know  2 = Yes |  |  |
| 10 | **Information or decisions made are clearly documented and disseminated to every member of the CREMA**  0 = Information or decisions are not documented & disseminated  1 = Information or decisions are sometimes documented but not disseminated  2 = Information or decisions are sometimes documented and disseminated to a few numbers of stakeholder groups  3 = Information or decisions are always documented & disseminated to a few numbers of stakeholder groups  4 = Information or decisions are sometimes documented & disseminated to majority of stakeholder groups  5 = Information or decisions are always documented & disseminated to majority of stakeholder groups |  |  |
| 11 | **The CREMA uses several means to communicate and disseminate information to its members**  ***Score:* ***0*** *if none is used;* ***0.5*** *each for each means that is used*   - Casual CRMC/CEC meetings - Monthly CRMC/CEC meetings - Annual General Meetings (AGM) - Community durbars/ outreaches - Phone calls - Radio and/or community information centres and data boards - House-to-house visits/delivery |  |  |
| 12 | **There are mechanisms for resolving conflicts among members and/or external stakeholders**  0 = No  1 = Don’t know  2 = Yes |  |  |

1. **Operational and institutional capacities**

| **#** | **Questions and response modalities** | **Score** | **Facilitator’s Notes/ Required Actions** |
| --- | --- | --- | --- |
| 1 | **The CREMA executives have clearly defined job descriptions that detail their primary duties, and they understand these duties**  0 = No  1 = Don’t know  2 = Yes |  |  |
| 2 | **Which statement best describes the capacity of the CREMA to own and lead the CREMA**  0 = The CREMA has no ability to mobilize its members to undertake activities and participate in district/national level decision-making processes related to natural resources management and to push for their own agenda  1 = The CREMA can mobilize its members to undertake activities and participate in district/national level decision-making processes related to natural resources management and to push for their own agenda but **with** external technical support (e.g., from NGOs)  2 = The CREMA can mobilize its members to undertake activities and participate in district/national level decision-making processes related to natural resources management and to push for their own agenda **without** external technical support (e.g., from NGOs) |  |  |
| 3 | **Has the CREMA been trained on a particular topic regarding natural resource protection/governance?**  0= No  1= Don’t know  2= Yes (List the topics of training)  - |  |  |
| 4 | **Which of the following skills/capacities can be demonstrated by the CREMA even without training?**  ***Score:* ***0*** *if no ability to apply skills;* ***0.5*** *for each skill that can be demonstrated*   - Ability to lead the CREMA - Ability to mobilize the community and facilitate events - Ability to speak up and advocate for its members - Ability to raise awareness on issues - Ability to establish and manage its own tree nursery - Ability to conduct patrol and report - Ability to monitor and report on activities - Ability to write proposals or raise funds - Ability to keep good records - Ability to plan budget and render accounts |  |  |
| 5 | **The CREMA has the equipment and material means to implement its activities (premises, computers, bicycles, motorbikes, boots, etc.)**  0 = No equipment at all  1 = Basic equipment but not enough to support its activities  2 = Has equipment sufficient to implement its activities  3 = Well-equipped and resourced to implement its activities |  |  |
| 6 | **The CREMA has equipment maintenance guidelines in place and are being implemented**  0 = The CREMA has no equipment maintenance plan  1 = The CREMA has an equipment maintenance plan, but it is not being implemented  2 - The CREMA has an equipment maintenance plan but is partially being implemented  3 - The CREMA has an equipment maintenance plan that is well-implemented |  |  |

1. **Best practices on cross-cutting themes**

| **#** | **Questions and response modalities** | **Score** | **Facilitator’s Notes/ Required Actions** |
| --- | --- | --- | --- |
| 1 | **Women, youth and other minority groups are represented in the CREMA governance structure.**  Women  0 = No representation  1 = Basic/less than 30%  2 = Moderate/between 30 to 50%  3 = Robust/above 50%  Youth  0 = No representation  1 = Basic/less than 30%  2 = Moderate/between 30 to 50%  3 = Robust/above 50%  Other (Pastoralists)  0 = No representation  1 = Basic/less than 30%  2 = Moderate/between 30 to 50%  3 = Robust/above 50% |  |  |
| 2 | **The ideas and views of women, youth and minority groups are considered and adopted in the CREMA’s resolutions**  0 = Don’t know  1 = Strongly disagree  2 = Disagree  3 = Neither agree or disagree  4 = Agree  5 = Strongly agree |  |  |
| 3 | **The CREMA is well recognized within the District Assembly, by the traditional authorities, the Forest Services and Wildlife Division**  0 = No  1 = Don’t know  2 = Yes, but not by all  3 = Yes, by all |  |  |
| 4 | **The CREMA has relationships and credibility with key technical experts, NGOs, private businesses, and other relevant stakeholders for building synergies**  0 = No  1 = Don’t know  2 = Yes |  |  |
| 5 | **The CREMA engages with and receives quality control checks from the Wildlife Division on the CREMA establishment and implementation process**  0 = Don’t know  1 = Strongly disagree  2 = Disagree  3 = Neither agree or disagree  4 = Agree  5 = Strongly agree |  |  |
| 6 | **The CREMA has a biodiversity patrol or monitoring team in place**  0 = No  1 = Don’t know  2 = Yes |  |  |
| 7 | **The CREMA has a biodiversity patrol or monitoring protocols/guidelines in place and being implemented**  0 = The CREMA has no monitoring protocol in place  1 = The CREMA has a monitoring protocol but is not being implemented  2 - The CREMA has a monitoring protocol but is partially being implemented  3 - The CREMA has a monitoring protocol and is well-implemented |  |  |
| 8 | **The CREMA conducts *regular* patrols to monitor and control illegal activities within the CREMA**  0 = No patrol/year  1 = Basic/less than 12 times/year  2 = Moderate/12 to 24 times/year  3 = Robust/Above 24 times/year |  |  |
| 9 | **The CREMA has an enterprise/venture that generates income for members, and its activities**  0 = No  1 = Don’t know  2 = Yes |  |  |
| 10 | **The CREMA has access to markets and links with private businesses for its products?**  0 = No  1 = Don’t know  2 = Yes |  |  |
| 11 | **The CREMA has long-term purchase agreements with buyers for its products**  0 = No  1 = Don’t know  2 = Yes |  |  |
| 12 | **The CREMA has other sources of funding (e.g., financial contribution of buyers, Moto King) to support its activities**  0 = No  1 = Don’t know  2 = Yes |  |  |
| 13 | **The CREMA has a bank account in place**  0 = No  1 = Don’t know  2 = Yes |  |  |
| 14 | **The CREMA has a conservation fund in place**  0 = No  1 = Don’t know  2 = Yes |  |  |
| 15 | **Which statement best describes the existence and implementation of the CREMA financial management plan?**  0 = The CREMA has no financial management plan  1 = The CREMA has a financial management plan but is not being implemented  2 = The CREMA has a financial management plan but is partially being implemented  3 = The CREMA has a financial plan and is well-implemented |  |  |

1. A fully functioning CRMC/CEC is one that holds meetings regularly and has a system for filing and documenting minutes and reports and provides feedback to its members as well as engages the relevant government authorities in the area. [↑](#footnote-ref-2)
