# Supplementary material for: Assessing CREMAs’ Capacity to Govern Landscape Resources in the Western Wildlife Corridor of Northern Ghana
Source: Environ Manage. 2025 Apr 4;75(5):1055–70. doi: 10.1007/s00267-025-02155-9 (PMC12033088; doi:10.1007/s00267-025-02155-9)
Supplement: Supplementary file 2 — Supplementary material 2 [file 267_2025_2155_MOESM2_ESM.docx]

**Supplementary material 2**

**Interview guide specific to resource persons, not members of CREMA committees**

| **Date of the assessment** |  |
| --- | --- |
| **Name of CREMA** |  |
| **Name of Community** |  |
| **Individual respondent** | Name: …………………………………………………………… Gender [ ] |
|  | Position: |
|  | Phone: |
| **Name(s) of facilitator(s)** |  |

| **#** | **Questions and response modalities** | **Comment/ Action needed** |
| --- | --- | --- |
| 1 | **Do you understand the CREMA concept?**  0 = Not at all  1 = A little  2 = Very well |  |
| 2 | **Are you familiar with the texts and rules governing the functioning of the CREMA?**  0 = Not at all  1 = A little  2 = Very well |  |
| 3 | **Which statement best describes your perception of the functioning of the CREMA?**  0 = The CREMA is not active at all  1 = I have no idea about the CREMA’s activities  2 = The CREMA is lowly active  3 = The CREMA is moderately active  4 = The CREMA is very active |  |
| 4 | **What capacities does the CREMA lack for better functioning? (List the five main ones)**  1:  2:  3:  4:  5: |  |
| 5 | **Do the members of the community participate in or support CREMA’s activities?**  0 = Don’t know  1 = Strongly Disagree  2 = Disagree  3 = Neither Agree or Disagree  4 = Agree  5 = Strongly Agree |  |
| 6 | **How can the participation/support of community members in the CREMA’s activities be improved?** |  |
