# Supplementary material for: Assessing CREMAs’ Capacity to Govern Landscape Resources in the Western Wildlife Corridor of Northern Ghana
Source: Environ Manage. 2025 Apr 4;75(5):1055–70. doi: 10.1007/s00267-025-02155-9 (PMC12033088; doi:10.1007/s00267-025-02155-9)
Supplement: Supplementary file 3 — Supplementary material 3 [file 267_2025_2155_MOESM3_ESM.docx]

**Supplementary material 3**

**Interview guide specific to off-site actors (District Assembly, Wildlife Division, etc.)**

| **Date of the assessment** |  |
| --- | --- |
| **Name of CREMA** |  |
| **Individual respondent** | Name: …………………………………………………………… Gender [ ] |
|  | Affiliation: |
|  | Position: |
|  | Phone: |
| **Name(s) of facilitator(s)** |  |

| **#** | **Questions and response modalities** | **Comment/ Action needed** |
| --- | --- | --- |
| 1 | **Are you familiar with the CREMA concept?**  0 = Not at all  1 = A little  2 = Very well |  |
| 2 | **Are you familiar with the texts and rules governing the functioning of CREMAs?**  0 = Not at all  1 = A little  2 = Very well |  |
| 3 | **Are other members of your team/organization/institution familiar with the CREMA concept and/or the rules governing the functioning of CREMAs?**  0 = Not at all  1 = A little  2 = Very well |  |
| 4 | **Which statement best describes your perception of the functioning of [name] CREMA?**  0 = The CREMA is not active at all  1 = I have no idea about the CREMA’s activities  2 = The CREMA is lowly active  3 = The CREMA is moderately active  4 = The CREMA is very active |  |
| 5 | **What capacities does [name] CREMA lack for better functioning? (List the five main ones)**  1:  2:  3:  4:  5: |  |
| 6 | **Your institution participates in or supports [name] CREMA’s activities.**  0 = Don’t know  1 = Strongly Disagree  2 = Disagree  3 = Neither Agree or Disagree  4 = Agree  5 = Strongly Agree |  |
| 7 | **What role does your institution play, or should it play in the CREMA functioning?**  **-**  **-**  **-** |  |
| 8 | **Does your institution have the necessary capacities to play its role vis-à-vis the CREMA and to support its functioning?**  1 = No capacities  2 = Basic capacities  3 = Enough capacities |  |
| 9 | **How can the support of your institution to the CREMA’s activities be improved?** |  |
